# Research to support evidence-informed decisions on optimizing gender equity in health workforce policy and planning

**DOI:** 10.1186/s12960-019-0380-6

**Published:** 2019-06-24

**Authors:** Neeru Gupta

**Affiliations:** University of New Brunswick, PO Box 4400, Fredericton, New Brunswick E3B 5A3 Canada

**Keywords:** Health workforce, Human resources for health, Gender equality, Occupational segregation, Leadership

## Abstract

Women constitute 70% of the global health and social care workforce, but important knowledge gaps persist to effectively support decision making to optimize gender equity. In this Editorial introducing a new thematic series on ‘Research to support evidence-informed decisions on optimizing gender equity in health workforce policy and planning,’ we are calling for submissions focusing on research concerning the monitoring, evaluation and accountability of human resources for health policy options through a gender equity lens. We are particularly interested to receive manuscripts advancing the innovative use of data and methodologies in the areas of occupational segregation, decent work, gender pay gap and gendered leadership in the health workforce that could be reproducible across different country contexts.

Countries and health agencies around the world are facing increasing strains to recruit and retain a health workforce aligned to current and future population health needs. Achieving this goal requires an overarching commitment to address potential imbalances and promote inclusive workplaces to help ensure a high performing health workforce. There is growing recognition that achieving gender inclusiveness and equity in health care entails transforming the systems within which women work, such as highlighted in a recent report [1] from the World Health Organization’s Gender Equity Hub [2].

Health services are often considered insufficiently responsive to women’s specific health needs, but they are also highly dependent on women as providers of care [3]. While women form the majority (70%) of the global health and social care workforce, important gaps persist to support evidence-informed decisions to optimize gender equity, notably in the areas of occupational segregation, decent work, gender pay gap and gender parity in leadership [1]. For example, males, including those in medical and other high-paying occupations, have long earned more than their female counterparts [4, 5], but only a few countries have legislative frameworks for public reporting of sex-disaggregated statistics on professional earnings and gender wage analyses [6]. Although the gender earnings gaps may be declining over time in some contexts, there remains a need for continued attention. Data from 21 countries indicate the average gender pay gap in the health workforce, after controlling for occupation and working hours, stands at 11% [7]. Such unexplained pay differentials may be attributed to a wide range of factors, including fewer opportunities for career advancement. Some research has found female nursing and midwifery personnel to be significantly less likely than their male counterparts to access in-service training [8]. It is widely acknowledged that women are under-represented in leadership roles. Less well known is what policy levers are most likely to lead to viable change. While sex (as a biological variable) is increasingly integrated in patient-oriented research focusing on health outcomes, sex-specific and especially gender-specific (socially constructed) considerations remain much less prevalent in research on human resources in health systems.

There is a dearth of data and evidence on the tipping points between gender-neutral versus gender-responsive human resources for health (HRH) policy options. Gender neutral refers to policies and programmes that are free of (explicit or implicit) reference to sex or gender, while gender responsive refers to being aware of and including gender as a socially important consideration [9]. Cross-national differences in workforce policies have been linked to gender responsiveness (or lack thereof) in factoring differential time use and work-life balance among women and men [10].

Research to inform gender-responsive policy guidance aims to analyse and accelerate opportunities for men, women, and gender-diverse people to overcome gender inequalities and (intentional or unintentional) biases in the health workforce. Policy guidance can achieve greater impact through better collection and use of data to assess how workforce initiatives may reflect or even intensify many of the social inequalities that health systems are meant to address and be immune from [3]. The use of sex-disaggregated data framed by gender analysis questions is a crucial starting point to understanding differences in the needs and experiences between male and female health workers for strengthening health systems [8, 11]. Given the urgent need for innovative actions to ensure the effective and ethical recruitment, management and retention of health workers across the working lifespan, relying on the status quo will not yield better performance.

In this thematic series on ‘Research to support evidence-informed decisions on optimizing gender equity in health workforce policy and planning,’ we are calling for submissions focusing on research concerning the monitoring, evaluation and accountability of HRH policy options through a gender equity lens. Possible sub-themes include, but are not limited to:

- Methods of determining pay for health workers as regards gender wage gaps
- Gender differences in workforce access to financial incentives for care delivery, such as performance bonuses
- Gender differences in pre-professional education and professional training opportunities
- Gender differences in access to social benefits among health workers, such as parental leave programmes or retirement pensions
- Effects of task-shifting from more specialized health workers (potentially traditionally male-dominated occupations) to less specialized workers (potentially female-dominated).

It is also increasingly recognized that the research process itself can perpetuate and reinforce gender inequalities [11]. For example, gender gaps in a health and health system research grant funding programme in Canada were recently attributed to less favourable assessments of women as principal investigators, rather than the quality of the proposed research [12]. Realization of the unintended consequences of (conscious or unconscious) gender biases in funding distribution by the national agency subsequently led to the cancellation of the given programme and reallocation of resources to other programmes that did not demonstrate bias in grant review.

Regardless of good intentions, some features of health systems may continue to be unintentionally disadvantageous for women. The series in *Human Resources for Health* aim to overcome the knowledge poverty on gender-responsive HRH policy and planning to support countries at all levels of development striving to achieve and sustain the global development goals of health workforce strengthening and gender equality. Authors wishing to have their manuscripts considered for inclusion in the new series should select this option when submitting to the journal. We are particularly interested to receive manuscripts advancing the use of data and methodologies that could be reproducible across different country contexts. In other words, manuscripts should describe how the methods can be used to build capacity among researchers, decision makers and other health system stakeholders to inform smarter HRH policies. Not all sex-disaggregated analyses will yield findings of inequalities, but it remains important that gender is considered as a core component to policy evaluation. Submissions with female first or joint-first authors are also highly encouraged. The ultimate goals are to foster excellence regarding the influence of sex and gender in HRH research, to actively support equal opportunities for HRH research publishing and to build communities of decision makers who are systematically integrating evidence-based gender considerations in health workforce policy and planning.

The World Health Organization’s Gender Equity Hub advocates for the improvement of data and evidence in the field [2], and therefore it supports this thematic series as part of the ongoing work on gender equity in the health workforce. Any opinion, finding and conclusion or recommendation expressed in this Editorial or any article published in this series are those of the authors and do not necessarily reflect the position of the WHO.

## Data Availability

Not applicable. No datasets were generated or analysed.
